# Assessment of biosecurity measures in broiler’s farms in the Suez Canal area – Egypt using a seasonal prevalence of Salmonellosis

**DOI:** 10.14202/vetworld.2020.622-632

**Published:** 2020-04-08

**Authors:** Essam S. Soliman, Mona S. Abdallah

**Affiliations:** 1 Department of Animal Hygiene, Zoonosis, and Animal Behavior, Faculty of Veterinary Medicine, Suez Canal University, Ismailia, 41522, Egypt; 2 Department of Avian and Rabbit Medicine, Faculty of Veterinary Medicine, Suez Canal University, Ismailia, 41522, Egypt

**Keywords:** biosecurity, broiler, Egypt, housing systems, *Salmonella*, seasons

## Abstract

**Background and Aim:**

Biosecurity practices are a must in broiler farms to reduce the risk of infectious agents. This study aimed to evaluate biosecurity measures in nine broiler farms in the Suez Canal area – Egypt with measuring the seasonal prevalence of salmonellosis.

**Materials and Methods:**

A cross-sectional study was conducted on randomly selected nine broiler farms of different housing systems based on the ventilation methods from March 2018 to April 2019. A total of 12,600 samples (6480 environmental, 4320 non-environmental, 1080 sera, and 720 live birds) were collected during four successive seasons.

**Results:**

Highly significant increases (p<0.01) were recorded in body weight gains in opened and closed-houses during summer; in food conversion ratios in opened-houses during winter and in closed-houses during winter and fall; in performance indices in opened-houses during summer and closed-houses during winter; and in live body weights, carcasses weights, liver, spleen, and bursa’s weights in opened-houses during spring and in closed-houses during fall. Highly significant increases (p<0.01) were recorded in total bacterial, Enterobacteriaceae, and *Salmonella* counts in opened-houses during spring and in closed-houses during summer, in *Salmonella* Typhi O and H, and *Salmonella* Paratyphi A and B sera titer in opened-houses during summer and closed-houses during fall. Biosecurity measures scored 34 out of 43 with an average salmonellosis prevalence of 6.0% in closed-house and 24 out of 43 with an average salmonellosis prevalence of 24.67% in opened-house broiler farms.

**Conclusion:**

Weak biosecurity measures in broiler houses (opened and closed) were not sufficient to prevent the entrance and multiplication of *Salmonella* spp. Disciplines, commitment, and regulations of biosecurity need to be enforced in broiler houses to prevent the introduction and spread of diseases.

## Introduction

Biosecurity can be described as principles, measures, and strategies that should be taken to prevent the introduction of a causative agent into a farm known as bioexclusion and to prevent the spread of a causative agent from one farm to another known as bioconfinement and biocontainment, as well as to prevent the spread of a causative agent inside the farm from one area to another known as biomanagement [1]. Successful biosecurity program depends initially on marking out boundaries of the production area inside the farm to ensure secure boundaries which represent the first line of defense against the introduction of infectious diseases [2]. Biosecurity programs in poultry farms should also consider some parameters, including the number of houses in each farm, the distance between houses, houses location and design, stocking density, interior arrangement, ventilation system, as well as, human, food, and material movement throughout the farm.

Poultry production depends on intensification and housing in closed confinement, which facilitates disease spread rapidly from one farm to another during an outbreak with economic devastation [3,4]. Inadequate biosecurity measures, absence and/or weak disease control measures and strategies [5], and poor management practices [6] contribute to high mortalities in poultry farms due to direct or indirect introduction and spread of some infectious and contagious pathogens such as infectious bursal disease (IBD) virus, Newcastle disease (ND) virus, and *Salmonella* spp. causing fowl typhoid and salmonellosis [7].

*Salmonella* Enteritidis normally inhabits the intestinal tract of birds, excreted in feces for months, and transmitted by insect, rodent, equipment, human being, soil, air, and water [8,9]. Once infection stabilized in a productive flock, it can transmit vertically and horizontally to infect other farms easily with zoonotic potential, especially *Salmonella* Typhimurium and *S*. Enteritidis [10]. The environment can act as a source of infection for salmonellosis, as *Salmonella* spp. can survive in the environment for a long period [11]. *Salmonella* spp. serological identification depends on using some commercial polyvalent antisera kits that contained a mixture of antibodies for determining the somatic (O) and flagellar (H) antigens [12].

The study aimed at evaluating the variable biosecurity measures installed in nine broiler farms with different housing systems based on the ventilation methods used (naturally ventilated or opened and artificially ventilated or closed-houses) in the Suez Canal area – Egypt using the seasonal prevalence of salmonellosis as an indicator for the successful application of these measures.

## Materials and Methods

### Ethical approval

The current protocol was reviewed and approved by the Scientific Research Ethics Committee of the Faculty of Veterinary Medicine, Suez Canal University, Ismailia, Egypt, with approval number (2019028).

### Study design

A cross-sectional study was conducted by visiting nine broiler farms in the Suez Canal area – Egypt. The nine farms were selected using systemic random sampling procedures, as recommended by Bell House[13]. Targeted farms’ selection was based on their geographical location in the three governorates overlook the Suez Canal area – Egypt (Port Said, Ismailia, and Suez governorates). Visits were carried out weekly for 13months (March 2018-April 2019). Farms under study were raising three different broiler breeds; are Ross 306 (in four farms, one located in Port Said, two located in Ismailia, and one farm located in Suez governorate), Cobb 500 (in two farms both located in Port Said governorate), and Hubbard classic broilers (in three farms, one located in Ismailia, and two farms located in Suez governorate).

Broilers in the nine farms were brooded and housed at optimal microclimatic temperature (received at 35°C, decreased gradually until a stable24-26°C was achieved by the end of the 3^rd^week with the latter comfort zone of 21-24°C). Broilers in the nine farms were supplied with a common continuous lighting regimen (23h lighting and 1h darkness) using white LED lights, as recommended by Soliman and Hassan[14]. Birds in the nine farms were given *ad libitum* access to drinking water and supplied with corn and soybean diet of the same constituents (El-Eman Co, El Sharkia, Egypt). The rations supplied to the broilers in the nine farms were containing 23% protein, 4.81% fat, 3.3% crude fiber, and 3000 kcal/kg energy in the starter ration and 21% protein, 5.89% fat, 3.5% crude fiber, and 3100 kcal/kg energy in the grower ration, as recommended by NRC [15] and Applegate and Angel[16].

Each farm followed a selective vaccination program, with a common baseline vaccination of birds against infectious bronchitis, IBD, and ND. The location, broilers’ breeds, housing system, number of units per farm, bird’s capacity in each unit, ventilation system and mechanism, feeding and watering systems, and lighting durations of each farm are listed in Table-1.

**Table-1:** Broiler farms’ basic information in the farms under evaluation for biosecurity measures.

Farm no.	Location	Broiler’s breed	Housing system	No. of units/Farm	Bird capacity/Unit	Ventilation system	Feed and watering system	Lighting program
1	Port said	Cobb	Deep litter	3	10000	Natural - Cross	Manual	23L:1D
2	Ismailia	Ross	Deep litter	5	5000	Natural - Cross	Manual	23L:1D
3	Ismailia	Hubbard	Deep litter	5	5000	Natural - Cross	Manual	23L:1D
4	Port Said	Cobb	Battery	5	10000	Natural - Cross	Manual	23L:1D
5	Suez	Hubbard	Deep litter	10	20000	Automated-Tunnel	Automated	23L:1D
6	Ismailia	Ross	Deep litter	5	10000	Natural-Cross	Manual	23L:1D
7	Suez	Hubbard	Slatted floor	20	20000	Automated-Tunnel	Automated	23L:1D
8	Suez	Ross	Slatted floor	20	20000	Automated-Tunnel	Automated	23L:1D
9	Port Said	Ross	Battery	5	10000	Natural-Cross	Manual	23L:1D

### Biosecurity evaluation and scoring system

Biosecurity measures in each of the nine farms were evaluated eventually according to certain characteristics as set by Wei and Aengwanich [17], including the following: Attraction of wild birds, prevention of wild birds, measures for farmworkers, measures for new poultry on the farms, measures for farm visitors, measures for vendors, measures for equipment and vehicles, water sources and water quality care, food sources, and environments surrounding farms including the distance between farms and roads, and other parts, type of poultry on farms, cleaning, and disinfection capacity on farms, measures from entry to poultry pens, and biosecurity system planning inside farms.

The nine farms that were previously identified and highlighted for regular weekly visits were evaluated and scored for each of the listed 14 characteristics according to a scale from 0 to 3, creating a top score of 43. The scoring was carried out weekly during the visits in reference to the compatibility of the interior arrangements and procedures enforced inside each farm regarding the characteristics set by Wei and Aengwanich [17] and was used for evaluation. Final average scores were calculated out of the values recorded during the 13months for each farm in reference to all the characteristics used for evaluations. The average means of salmonellosis (mean ±SE) are displayed inTable-2 in relation to the different housing systems, and seasonal variation along with their interactions, as well as average scores of biosecurity measures for each farm are displayed in Table-3.

**Table-2:** *Salmonella* Serotypes titer (Mean ±SE) in sera collected from broilers raised on different housing systems during different seasons.

Housing system	*Salmonella* Serotype titer				*Salmonella* prevalence %

	O	H	AH	BH	
Overall means of the housing system					
Opened-house	157.2^a^±2.83	163.9^a^±2.99	110.6^a^±1.44	113.6^a^±1.57	24.6^a^±0.45
Closed-house	104.8^b^±4.01	104.6^b^±4.23	81.1^b^±2.03	80.0^b^±2.23	6.0^b^±0.05
p-value	**0.016**	**0.009**	**0.001**	**0.000**	**0.001**
Overall means of the seasons					
Summer	141.8^a^±2.91	138.1^a^±5.12	100.4^a^±2.49	105.7^a^±2.73	15.6^a^±0.06
Fall	133.8^a^±2.88	138.7^a^±3.17	93.3^a^±1.94	93.1^a^±1.55	13.8^b^±0.12
Winter	127.1^b^±3.46	132.4^a^±5.18	95.3^a^±1.66	96.0^a^±2.34	9.6^c^±0.08
Spring	121.4^b^±2.65	127.8^a^±4.14	94.4^a^±1.93	92.4^a^±3.22	5.2^d^±0.06
p-value	**0.025**	**0.516**	**0.624**	**0.725**	**0.002**
Housing system versus seasonal variation interactions					
Opened-house					
Summer	177.0^a^±7.17	174.0^a^±6.97	119.1^a^±4.15	131.5^a^±5.33	29.2^a^±0.08
Fall	152.2^b^±6.35	161.1^b^±6.68	106.6^b^±3.56	106.2^c^±3.01	28.6^a^±0.02
Winter	150.2^b^±6.29	158.2^b^±6.78	110.6^b^±3.10	112.0^b^±3.48	22.0^b^±0.01
Spring	149.5^b^±6.67	162.3^b^±7.32	106.2^b^±3.01	104.8^c^±3.16	19.3^c^±0.05
p-value	**0.092**	**0.182**	**0.123**	**0.002**	**0.000**
Closed-house					
Summer	106.6^b^±3.99	102.2^b^±3.79	81.7^a^±1.24	80.0^a^±0.00	9.5^a^±0.03
Fall	115.5^a^±5.22	116.4^a^±6.08	80.0^a^±0.00	80.0^a^±0.00	5.0^b^±0.01
Winter	104.0^b^±3.88	106.6^b^±5.05	80.0^a^±0.00	80.0^a^±0.00	5.3^b^±0.02
Spring	93.3^c^±3.16	100.3^b^±3.16	82.6^a^±0.00	80.0^a^±0.00	4.3^b^±0.01
p-value	**0.023**	**0.098**	**0.325**	**0.815**	**0.000**

Means carrying different superscripts in the same column are significantly different at (p≤0.05) or highly significantly different at (p<0.01). Means carrying the same superscripts in the same column are non-significantly different at (p<0.05).O=*Salmonella* Typhi O, H=*Salmonella* Typhi H, AH=*Salmonella* Paratyphi A, BH=*Salmonella* Paratyphi B, opened=naturally ventilated houses, Closed=artificially ventilated houses, SE=Standard error

**Table-3:** The evaluation system for the biosecurity programs and the prevalence of salmonellosis in farms under study.

Evaluation items	F1	F2	F3	F4	F5	F6	F7	F8	F9
Av. Prevalence of salmonellosis %	29	25	27	23	5	18	7	6	26
Attraction of wild birds	3	2	2	1	0	3	0	0	2
Prevention of wild birds	0	1	2	2	3	0	3	3	2
Measures for farm workers	1	1	1	2	2	1	1	2	2
Measures for new poultry on the farms	2	2	1	2	3	2	3	3	3
Measures for farm visitors	1	2	1	2	3	1	3	3	2
Measures for vendors	1	2	1	2	3	1	2	2	1
Measures for equipment and vehicles	1	2	2	2	2	1	2	2	2
Water sources and water quality care	2	2	2	3	3	2	3	3	2
Food sources	1	2	2	3	3	1	3	3	2
Environments surrounding farms: Distance between farms and roads, and other parts	2	2	1	2	3	2	3	3	2
Type of poultry on farms	1	2	2	2	3	1	3	3	3
Cleaning and disinfection capacity on farms	2	2	1	2	2	2	2	3	2
Measures from entry to poultry pens	2	2	2	2	3	2	3	2	2
Biosecurity system planning inside farms	1	2	2	2	2	1	2	3	2
Total score out of 43	20	26	22	29	35	20	33	35	29

Zero-point was the lowest score and 3 points were the highest score for each point out of the 14 points for evaluating the biosecurity measures, F=farm. F1, F2, F3, F4, F6, and F9 are naturally ventilated (opened-house) farms with an average biosecurity score of 24 out of 43 and average salmonellosis prevalence 24.67%; F5, F7, and F8 are artificially ventilated (closed-house) farms with an average score of 34 out of 43 and average salmonellosis prevalence 6.0%

### Sampling

A total of 12600samples were collected during the four seasons from the nine farms, including 6480 environmental samples (litter, water, walls, fans, feeders, and waterers swabs), 4320 non-environmental samples (liver, spleen, duodenum of the intestine, and breast muscle), 1080 blood samples for sera separation, and 720 live birds were collected by the end of the studied cycles for carcass quality evaluation. Blood samples were transferred to the laboratory in a dry ice box and centrifuged at 2500rpm for 20min for sera separation. Clear non-hemolyzed sera were stored at −20°C until used for the serological identification of *Salmonella* spp.

Birds collected for carcass evaluation were transported using conditioned trucks. On their arrival, birds were rested for 3h then slaughtered in the institutional experimental slaughter room. Liver, spleen, heart, and bursa were extracted, weighed, and expressed as (g/kg), and the carcasses were weighed after de-feathering and evisceration and expressed as (g), as recommended by Soliman *et al*. [18]. The carcasses were hygienically disposed of after weighing and sampling using burial technique in reference to the lining of the burial bits with lime.

Environmental swabs, as well as, non-environmental samples were collected and placed in 9ml buffered peptone water, preserved in a dry ice box supplied with gel bags to maintain the samples and retard any biological changes, and transferred to the laboratory for bacteriological assessment considering a transportation time that did not exceed 2-3h.

### Performance indices (PI)

Live body weights (LBW) expressed by grams (g) were measured based on weighing representative samples of birds in each farm, the representative sample sizes were calculated using a simple random sampling design [19] with an expected error 5% using the following formula:


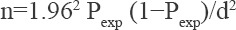


Where n=required sample size, P_exp_=expected prevalence, and d=desired absolute precision. Approximately 286 out of each 1000 birds in each farm were weighed to obtain representative and accurate measures. Feed intakes (FI, expressed by g) were calculated by dividing the total amounts of ration consumed by birds in each building by the actual number of birds housed in this building. Bodyweight gains (BWG, expressed by g), feed conversion ratio (FCR, expressed by %), and (PI, expressed as a ratio) were calculated, as recommended by Soliman and Hassan[20].

### Serological analysis of *Salmonella* spp

Sera were used in a serological quantitative antigen-antibody micro-well agglutination test [21,22]. The test aims at detecting *Salmonella* Typhi immunoglobulin M against the somatic antigen (O), flagellar antigen (H), *Salmonella* Paratyphi A (AH), and *S*. Paratyphi B (BH). The degree of agglutination was observed and quantified.

### Bacteriological examination

Litter samples were prepared by weighing and adding 3g into 27ml physiological saline and filtered, as recommended by Soliman *et al*. [23]. Non-environmental organs such as the liver, spleen, duodenum of the intestine, and breast muscles were added to 0.1% sterile buffered peptone water, homogenized using a stomacher (Lab. Blender 400, Seward Lab., London). Litter filtrate, water samples, environmental swabs, and non-environmental homogenized extract were prepared according to APHA [24] using ten-fold serial dilutions up to 10^−6^ to cover the expected range of samples contamination, which could be easily counted.

Total bacterial count (TBC) onto standard plate count agar (SPC, APHA dehydrated Thermo Scientific^™^ Oxoid^™^ CM0463B, weight 500g) at 37°C for 24h, total Enterobacteriaceae count (TEC) onto Eosin methylene blue agar (EMB, Modified Levine Thermo Scientific^™^ Oxoid^™^ CM0069B, weight 500g) at 37°C for 24h for detecting the ideal metallic green colonies, and total *Salmonella* count (TSC) onto CHROMagar^™^ *Salmonella* (BD BBL^™^ CHROMagar^™^ *Salmonella* READY-TO-USE Plated Media) at 37°C for 24h for detecting the ideal pink colonies were applied using drop plate technique, according to Kim and Lee [25]. Plates showed that 30-300 colony-forming units (CFU) were counted using the Darkfield colony counter (R164109 Reichert-Jung Quebec Darkfield 3325 Colony Counter) [26].

### Statistical analysis

Statistical analysis was carried out using the Statistical Package for the Social Sciences (SPSS), IBM SPSS statistics version-20 [27]. The obtained data were analyzed statistically using multifactorial Analysis of Variance (Two-way ANOVA) investigating the effects of the housing systems based on ventilation methods and seasons along with their interactions. The interaction effects of the housing systems (closed and opened) and the seasons (summer, fall, winter, and spring) were displayed in the tables along with the overall and main effects. The statistical model was summarized as follow:


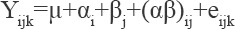


Where Y_ijk_ was the measurement of the dependent variables; µ was overall mean; α_i_ was the fixed effect of the housing system; β_j_ was the fixed effect of the season; (αβ)_ij_ was the interaction effect of the housing systems by seasons; e_ijk_ was the random error. The significant levels were expressed as highly significant at p<0.01, significant at p≤0.05, and non-significant at p>0.05. The bacterial counts were transferred into logarithmic numbers as well as, the average scores for the nine farms understudy were calculated using Microsoft Excel 2016.

## Results

### Performance traits

Increases in weight gains and PI of broilers raised in closed-houses were highly significant (p<0.01) and the increases in FI and FCRs of broilers raised in opened-houses were also highly significant (p<0.01; Table-4), there were no significant differences in the weight gains among the four seasons (Table-4). With regard to FI, highly significant increases (p<0.01) were observed during winter, spring, fall, and summer, respectively (Table-4). Increases in FCRs were highly significant (p<0.01) as observed during winter, fall, spring, and summer, respectively, with no significant differences between FCRs during fall and spring seasons (Table-4). PI revealed highly significant increases (p<0.01) during spring, summer, fall, and winter seasons, respectively, with no significant differences between PI during fall and winter seasons (Table-4).

**Table-4:** Performance traits (Mean±SE) in broilers raised on different housing systems during different seasons.

**Housing system**	Performance traits			

	WG/g	FI/g	FCR (%)	PI
Overall means of the housing system				
Opened-house	341.7^b^±2.91	557.7^a^±0.95	1.66^a^±0.01	5.30^b^±0.06
Closed-house	397.1^a^±4.11	463.1^b^±1.34	1.20^b^±0.02	8.57^a^±0.09
p-value	**0.001**	**0.005**	**0.002**	**0.000**
Overall means of the seasons				
Summer	378.0^a^±1.22	495.4^d^±1.62	1.34^c^±0.02	7.40^b^±0.11
Fall	369.0^a^±2.13	506.5^c^±0.93	1.42^b^±0.01	6.93^c^±0.09
Winter	361.6^a^±1.33	526.1^a^±0.99	1.53^a^±0.01	6.50^c^±0.08
Spring	369.1^a^±2.11	513.5^b^±1.22	1.43^b^±0.01	9.63^a^±0.12
p-value	**0.532**	**0.001**	**0.000**	**0.023**
Housing system versus seasonal variation interactions				
Opened-house				
Summer	352.8^a^±4.23	531.8^d^±1.90	1.52^c^±0.02	5.91^a^±0.13
Fall	341.6^b^±5.82	548.7^c^±2.23	1.62^b^±0.03	5.34^b^±0.21
Winter	330.5^c^±2.31	584.6^a^±3.21	1.82^a^±0.02	4.71^c^±0.31
Spring	342.1^b^±5.66	565.7^b^±0.98	1.67^b^±0.02	5.25^b^±0.14
p-value	**0.037**	**0.000**	**0.010**	**0.038**
Closed-house				
Summer	403.2^a^±8.23	459.0^a^±2.21	1.15^b^±0.03	8.89^a^±0.19
Fall	396.4^b^±6.21	464.3^a^±2.69	1.21^a^±0.04	8.52^a^±0.22
Winter	392.8^b^±5.41	467.7^a^±2.65	1.24^a^±0.04	8.28^b^±0.17
Spring	396.2^b^±4.32	461.3^a^±3.41	1.19^ab^±0.02	8.61^a^±0.19
p-value	**0.867**	**0.662**	**0.056**	**0.223**

Means carrying different superscripts in the same column are significantly different at (p≤0.05) or highly significantly different at (p<0.01). Means carrying the same superscripts in the same column are non-significantly different at (p<0.05).WG=Weight Gain, FI=Feed Intake, FCR=Feed Conversion Ratio, PI=Performance Index, Opened=naturally ventilated houses, Closed=artificially ventilated houses, SE=Standard error

BWGs interactions, as recorded in Table-4, showed highly significant increases (p<0.01) during summer in both opened-and closed-house broiler farms. FIs interactions revealed, in Table-4, highly significant increases (p<0.01) during winter in opened-houses, and no significant differences were recorded during all seasons in closed-house broiler farms.

FCRs’ interactions recorded highly significant increases (p<0.01) during winter in opened-house broiler farms, and during winter and fall with no significant difference in closed-house broiler farms (Table-4). Meanwhile, the PI interactions in Table-4 revealed highly significant increases (p<0.01) during summer and winter in opened-and closed-house broiler farms, respectively.

### Carcass quality

Overall means revealed in Table-5, highly significant increases (p<0.01) of LBW, carcass weights, spleen, and heart ratios in closed-house broiler farms, highly significant increases (p<0.01) of liver ratios in opened-house broiler farms, and no significant differences between the two housing systems in bursa’s ratios. LBW and heart ratios (Table-5) revealed highly significant increases (p<0.01) during fall, spring, summer, and winter seasons, respectively. Carcasses weights, spleen, and bursa ratios in Table-5 revealed highly significant increases (p<0.01) during fall, spring, summer, and winter, respectively, with no significant differences between carcasses weights during fall and spring seasons. The liver ratios (Table-5) revealed highly significant increases (p<0.01) during spring, fall, summer, and winter seasons, respectively.

**Table-5:** Live body weight and carcass quality characteristics (Mean±SE) in broilers raised on different housing systems during different seasons.

Housing system	LBW/ =g	Carcass wt./g	Organs/Carcass ratio			

			Liver	Spleen	Heart	Bursa
Overall means of the housing system						
Opened-house	1749^b^±4.6	1322^b^±5.6	3.2^a^±0.02	0.14^b^±0.00	0.90^b^±0.01	0.08^a^±0.00
Closed-house	2074^a^±6.3	1756^a^±8.0	3.1^b^±0.02	0.18^a^±0.01	1.01^a^±0.01	0.08^a^±0.00
p-value	**0.002**	**0.000**	**0.005**	**0.001**	**0.004**	**0.056**
Overall means of the seasons						
Summer	1935^c^±2.5	1552^b^±5.6	3.2^c^±0.01	0.16^b^±0.00	0.86^c^±0.02	0.08^b^±0.00
Fall	2015^a^±3.6	1590^a^±9.8	3.3^b^±0.02	0.17^a^±0.00	1.14^a^±0.01	0.09^a^±0.01
Winter	1701^d^±1.2	1429^c^±2.3	2.8^d^±0.02	0.12^c^±0.01	0.76^d^±0.02	0.06^c^±0.00
Spring	1995^b^±5.6	1586^a^±5.8	3.4^a^±0.03	0.17^a^±0.00	1.06^b^±0.01	0.09^a^±0.01
p-value	**0.005**	**0.012**	**0.001**	**0.009**	**0.006**	**0.009**
Housing system versus seasonal variation interactions						
Opened-house						
Summer	1787^b^±11.0	1345^c^±12.8	3.2^c^±0.03	0.13^c^±0.00	0.76^c^±0.01	0.07^c^±0.00
Fall	1844^b^±15.1	1379^b^±15.3	3.4^b^±0.03	0.15^b^±0.00	1.09^a^±0.03	0.08^b^±0.00
Winter	1497^d^±6.7	1168^d^±12.8	2.9^d^±0.04	0.10^c^±0.00	0.74^c^±0.01	0.07^c^±0.00
Spring	1866^a^±9.5	1396^a^±11.6	3.5^a^±0.03	0.16^a^±0.00	1.01^b^±0.01	0.09^a^±0.00
p-value	**0.000**	**0.000**	**0.006**	**0.000**	**0.001**	**0.004**
Closed-house						
Summer	2083^c^±6.25	1758^c^±8.99	3.1^c^±0.03	0.18^c^±0.00	0.96^c^±0.02	0.08^b^±0.00
Fall	2185^a^±5.97	1801^a^±8.71	3.3^a^±0.04	0.20^a^±0.00	1.20^a^±0.02	0.09^a^±0.00
Winter	1905^d^±5.70	1689^d^±7.45	2.7^d^±0.03	0.13^d^±0.00	0.78^d^±0.01	0.05^c^±0.00
Spring	2123^b^±5.78	1777^b^±8.53	3.2^b^±0.03	0.19^b^±0.00	1.11^b^±0.02	0.09^a^±0.00
p-value	**0.006**	**0.000**	**0.000**	**0.000**	**0.004**	**0.009**

Means carrying different superscripts in the same column are significantly different at (p≤0.05) or highly significantly different at (p<0.01). Means carrying the same superscripts in the same column are non-significantly different at (p<0.05).LBW=Live Bodyweight, Carcass wt=Carcass weight, opened=naturally ventilated houses, Closed=artificially ventilated houses, SE=Standard error

LBW, carcasses weights, liver; spleen, and bursa’s ratios recorded highly significant increases (p<0.01), as shown in Table-5, during spring and fall seasons in opened-and closed-house broiler farms, respectively. The heart ratios revealed highly significant increases (p<0.01) during the fall in opened-and closed-house broiler farms.

### Bacterial counts

TBCs revealed highly significant increases (p<0.01) in environmental and non-environmental samples collected from opened-house broiler farms (4.71 CFU/ml) compared to those from closed-house broiler farms (4.32 CFU/ml). TECs revealed highly significant increases (p<0.01) in environmental and non-environmental samples collected from opened-house (2.87 CFU/ml) compared to those from closed-house broiler farms (1.77 CFU/ml). TSC overall means revealed highly significant increases (p<0.01) in environmental and non-environmental samples collected from opened-house broiler farms (0.51 CFU/ml) compared to those from closed-house broiler farms (0.12 CFU/ml).

TBCs revealed highly significant increases (p<0.01) in environmental and non-environmental samples collected during spring, winter, summer, and fall seasons (4.57, 4.50, 4.49, and 4.41 CFU/ml, respectively). TECs revealed highly significant increases (p<0.01) in environmental and non-environmental samples collected during summer, winter, spring, and fall seasons (4.57, 4.50, 4.49, and 4.41 CFU/ml, respectively) with no significant differences between TEC during winter and spring seasons. TSCs revealed highly significant increases (p<0.01) in environmental and non-environmental samples collected during summer, spring, winter, and fall seasons (0.38, 0.38, 0.36, and 0.35 CFU/ml, respectively) with no significant differences between TSCs during summer and spring seasons.

**Figure-1:**
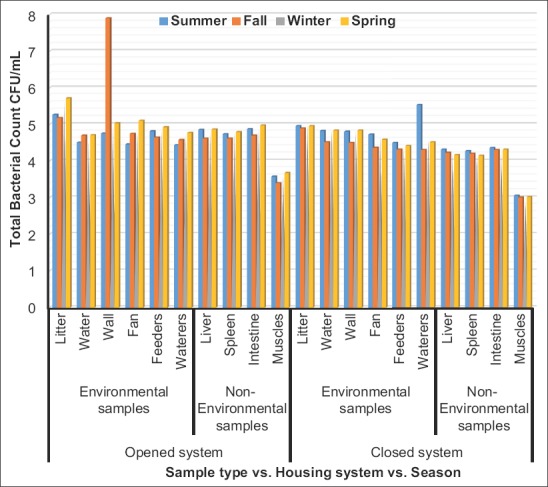
Logarithm total bacterial counts (Mean±Standard error colony-forming units/ml) in environmental and non-environmental samples collected from different housing systems during different seasons.

**Figure-2:**
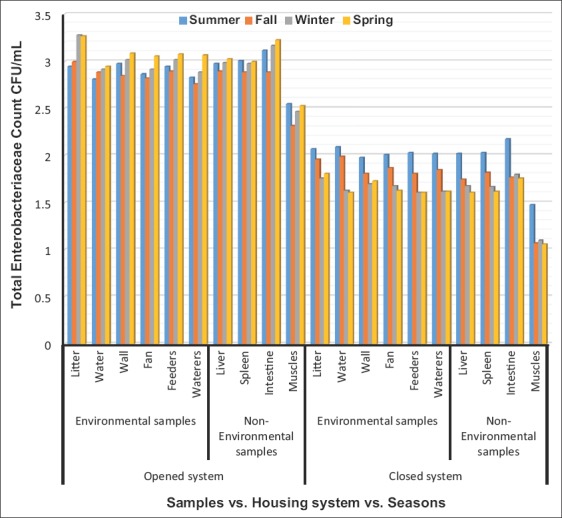
Logarithm total Enterobacteriaceae counts (Mean±Standard error colony-forming units/ml) in environmental and non-environmental samples collected from different housing systems during different seasons.

Total bacterial and Enterobacteriaceae counts interactions, in Figures-1 and 2, revealed highly significant increases (p<0.01) in the litter, water, fans, feeders, waterers, swabs, liver, spleen, intestine, and breast muscles collected from opened-house broiler farms during spring, and in the wall swabs during fall and spring, respectively. Highly significant increases (p<0.01) were recorded in TBC and TEC of litter, water, walls, fans, feeders, waterers swabs, liver, spleen, intestine, and breast muscles of closed-house broiler farms during summer compared to other seasons.

**Figure-3:**
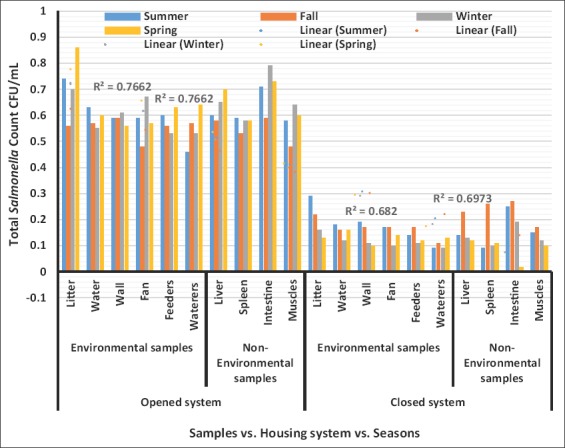
Logarithm total *Salmonella* counts (Mean± Standard error colony-forming units/ml) in environmental and non-environmental samples collected from different housing systems during different seasons.

TSCs (Figure-3) revealed highly significant increases (p<0.01) in the litter, water, walls, feeders, waterers swabs, liver, intestine, and breast muscle during spring in opened-house, and summer in closed-house broiler farms. Meanwhile, in Figure-3, highly significant increases (p<0.01) were observed during winter and summer in fan swabs, and during spring and fall in the spleen ratios of opened-and closed-house broiler farms, respectively.

### *Salmonella* serotype and prevalence

*S*. Typhi O and H and *S*. Paratyphi A and B titer revealed in Table-2 highly significant increases (p<0.01) in opened-house compared to closed-house broiler farms. *S*. Typhi O (Table-2) revealed highly significant increases (p<0.01) in summer, fall, winter, and spring, respectively, with no significant differences during summer and fall seasons and between winter and spring seasons. *S*. Typhi H and S. Paratyphi A and B titer revealed in Table-2 no significant differences between the four seasons.

*S*. Typhi O and H, as well as, *S*. Paratyphi A and B titer revealed, in Table-2, highly significant increases (p<0.01) in opened-house broiler farms during summer seasons and in closed-house broiler farms during fall seasons. *Salmonella* spp. prevalence revealed highly significant increases (p<0.01) during summer, fall, winter, and spring, respectively. The calculation of the average prevalence percent of salmonellosis in both types of broiler farms understudy (Table-2) showed that the prevalence of salmonellosis in the closed-house broiler farms (6.0%) was significantly lower (p<0.01) compared to that in the opened-house broiler farms (24.67%).

### Biosecurity measures status

A total score was given for each farm as a sum for all individual average scores for the listed evaluation items, as shown in Table-3. The calculated average score for the closed-house broiler farms was 34 out of 43 and for the opened-house broiler farms was 24 out of 43. The results revealed higher levels of commitment and disciplines of biosecurity measures in closed-house compared to opened-house broiler farms.

## Discussion

Poultry production worldwide and in Egypt depends on intensification in naturally ventilated (opened) and artificially ventilated (closed) housing systems associated with different floor systems such as deep litter, battery, and slatted floor systems with or without an all-in-all out policy. Broilers in the commercial and small yard production remain inside their raising or growing and finishing houses for the entire cycle, then removed for slaughtering or transferred to another egg-laying facility [28]. Chapman and Jeffers [29] classified the transportation conditions to the slaughterhouses and the movement of birds to other buildings for egg production as suitable conditions contribute to lowering broiler’s resistance and creating a perfect media and circumstances that enhance the introduction and establishment of infectious agents, colonize broilers, and establish a cycle of infection unless there are enough preventive measures installed to prevent the introduction and spread of these pathogens.

Small scale and backyard poultry production in Egypt has become a profitable industry for many reasons including minimum maintenance requirements, profitable and fast financial outcome, coverage of a gap in public demand for animal protein, easy marketing, provision of high-quality fertilizer, and easy control through the application of a small number of preventive measures [30]. Total household and backyard poultry population in Egypt is undefined, and all the authorized and available governmental data referred to the financial investment sizes without exact numbers, but according to Helal *et al*. [31], about 9.5 million poultry are raised in small and confined places with little or no biosecurity measures. ElMasry *etal*.[32] reported a significant role in the backyard and household production in the spread and transmission of many infectious and zoonotic diseases.

The prevalence of salmonellosis in the current study revealed a significantly higher prevalence in the opened-house compared to closed-house broiler farms without neglecting the biosecurity measures installed in each farm, and the favorable macroclimatic condition predominated to stimulate *Salmonella* spp. growth and survival. Rothrock *et al*. [33] indicated that the poultry industry has been incriminated in many salmonellosis outbreaks among poultry and human populations worldwide with special reference to *Salmonella enterica* serovar Enteritis and *S. enterica* serovar Typhimurium. Fasanmi *et al*. [34] stated that the persistence of pathogens such as avian influenza H5N1 and *Salmonella* in certain geographical areas is attributed to poor biosecurity measures in the poultry farms and live bird markets.

Soliman *et al*. [35] have earlier conducted an epidemiological survey and isolated *Escherichia coli, Salmonella* spp.*, Proteus vulgaris, Shigella* spp*., Pseudomonas aeruginosa*, and *Streptococcus faecalis* from 416 environmental samples collected during the period January 2008-July 2008 from commercial poultry farms in Ismailia and Zagazig governorates – Egypt, and from 266 environmental and non-environmental samples collected during the period September 2008-January 2009 by Alabama State Veterinary Diagnostic Laboratory – USA. They attributed these large proportions of microbial contaminants to the lack of sufficient hygienic and biosecurity measures in these farms. Our results were not consistent with those recorded by Wierup *et al*. [36] who compared the occurrence of salmonellosis during 2007-2015 in broiler and laying hen in outdoor and indoor production under the influence of the synchronized conditions for prevention and control of *Salmonella* spp. in Sweden. They found a very small annual incidence of *Salmonella* spp. in outdoor production (0-2.0%) similar to that of indoor production (0-1.3%), they attributed this presence to the horizontal and vertical transmission means of *Salmonella* spp.

*S*. Typhi O and H detection with higher prevalence in opened-house broiler farms in our study might be attributed to the misapplication of biosecurity main disciplines as disinfection procedures. Results agreed with those of Ahmed *et al*. [37] who investigated the efficiency of 125 and 250ml chlorine dioxide 2000ppm against *E. coli* and *Salmonella* spp. They revealed a significant reduction in both types of microorganisms in normal microclimatic conditions.

Biosecurity has been defined as “informed common sense,” meaning obligations to learn principles and regulations of biosecurity, and mixed with common management practices. Discipline, accountability, and belief are the main three pillars on which the biosecurity program relies. The final average scores calculated in the current study for each of the nine farms implemented an average of 34 out of 43 for closed-house and 24 out of 43 for opened-house broiler farms, meaning higher commitment in closed-house broiler farms toward the application of biosecurity measures compared to opened-house broiler farms. The results were synchronized with those recorded by Aiyedun *et al*. [38] who investigated biosecurity status in some broiler farms depending on the presence of a fence, traffic signals, dead bird disposal methods, usage of protective clothes, access of wild birds, and rodents. They explained that the misapplication of basic hygienic and biosecurity measures contributed to serious consequences on birds’ and human health.

In the current study, cleaning and disinfection programs were observed to ensure applying biosecurity measures announced on each farm. Dry cleaning was applied to remove loose dirt, followed by wet cleaning using detergent and water under pressure, with paying attention for corners, joints, and fissures in walls and floors. The most predominating detergents that were used in the nine farms were quaternary ammonium compounds or a mixture of aldehydes and quaternary ammonium compounds. Disinfection processes were carried out using sodium hydroxide followed by formaldehyde spray in opened-house broiler farms, and the same procedures were used in addition to sodium hypochlorite as an additional step in closed-house broiler farms. Cleaning and disinfection procedures in some farms understudy were effective to minimize the entrance of *Salmonella* spp. The results are in agreement with those of Klosha *et al*. [39] who reported that the application of risk-oriented hygiene contributed minimization of public health risk from *Salmonella* spp., and 66% reduction in salmonellosis incidence can be achieved through intensive cleaning. Soliman *et al*. [40] evaluated the disinfection regimens in some commercial poultry farms located in Ismailia and Zagazig governorates – Egypt. They recovered some bacterial microorganisms such as *Klebsiella oxytoca, Citrobacter* spp*., P. vulgaris, E. coli, P. aeruginosa, Shigella* spp*., Salmonella* spp*., S. faecalis, Staphylococcus aureus*, and *Streptococcus pneumoniae*, and some fungal organisms such as *Yeast* spp*., Candida albicans*, *Aspergillus flavus, Aspergillus niger, Aspergillus nidulans, Mucor*, and *Penicillium* spp. They attributed the recovery of these organisms to the failure of disinfection regimens that were followed in these farms.

The recorded results were also consistent with those of Luyckx *et al*. [41], who evaluated the efficacy of four cleaning programs to reduce *E. coli* and *Salmonella* spp. infection in 12 broiler houses. They recorded a significant reduction in total aerobic flora and Enterococcus spp. counts in swabs after the cleaning process with no differences between protocols. Course [42] investigated disinfection status as a key role in biosecurity program in the commercial broiler farms in Ontario, and he revealed that efficient disinfection procedures can be detrimental for many pathogens such as *E. coli, Clostridium perfringens*, and *S. enterica*. Soliman *et al*. [43] found that cleaning and disinfection procedures using the recommended disinfectants’ concentration were not able to control microbial growth and subsequent contamination with some zoonotic enteric pathogens such as *E. coli* and *Salmonella* spp.

Our results agreed with those recorded by Limbergen *et al*. [44], who quantified the biosecurity levels in broiler farms using a risk-based weighted scoring system, consisted of an external scoring system with eight subcategories and internal scoring system with three subcategories. They reported that broiler farms had internal biosecurity 76.6 and external biosecurity 68.4; the results indicated the presence of wide variation for both levels of biosecurity, and the great possibility for improving biosecurity level of each farm.

## Conclusion

Biosecurity measures were enforced with a higher degree of success in artificially ventilated (closed-house) compared to naturally ventilated (opened-house) broiler farms. Needless to say, installing strict biosecurity measures in both types of broiler houses were not enough to prevent the growth and multiplication of *Salmonella* spp. The lack of commitment to the adopted measures contributed a breach through which microorganisms can enter, stabilize themselves, develop infectious and contagious diseases, and spread from one area to another inside the same farm or to other farms easily.

Control of salmonellosis in broiler farms regardless of the housing system, location, holding capacity, and ventilation system depend mainly on strict measures including good hygienic measures, early detection, and effective cleaning and disinfection program.

## Authors’ Contributions

ESS designed the cross-sectional study and sampling design, visited farms weekly during the study period, scored each farm according to the installed measures, collected samples, participated in samples analysis, interpreting results, and in writing of the manuscript. MSA participated in sample analysis, interpreting results, and writing of the manuscript.

## References

[1] Gussem M.D, Meddelkoop K.V, Mullen K.V, Veer E.V (2013). Broiler Signals:A Practical Guide for Broiler Focused Management.

[2] Biosecurity Act (2015). New South Wales Biosecurity Manual, Proposed Biosecurity Regulation.

[3] Aengwanich W, Boonsorn T, Srikot P (2014). Intervention to improve biosecurity system of poultry production clusters (PPCs) in Thailand. Agriculture.

[4] Sayeed M.A, Smallwood C, Imam T, Mahmud R, Hasan R.B, Hasan M, Anwer M.S, Rashid M.H, Hoque M.A (2017). Assessment of hygienic conditions of live bird markets on avian influenza in Chittagong metro, Bangladesh. Prev. Vet. Med.

[5] Conan A, Goutard F.A, Sorn S, Vong S (2012). Biosecurity measures for backyard poultry in developing countries:A systematic review. BMC Vet. Res.

[6] Rajagopal R, Mini M (2013). Outbreaks of salmonellosis in three different poultry farms of Kerala, India. Asian Pac. J. Trop. Biomed.

[7] Eltholth M.M, Mohamed R.A, Elgohary F.A, Elfadl E.A (2016). Assessment of biosecurity practices in broiler chicken farms in Gharbia governorate, Egypt. Alex. J. Vet. Sci.

[8] Dar M.A, Ahmad S.M, Bhat S.A, Ahmed R (2017). *Salmonella* Typhimurium in poultry:A review. Worlds Poult. Sci. J.

[9] Bhunia A.K (2018). Food Borne Microbial Pathogens:Mechanisms and Pathogenesis.

[10] Nirmala T.V, Reddy A.D, Sree E.K, Subbaiah K.V, Raju G.S, Reddy R.V.S (2018). Salmonellosis in poultry:A case report. Int. J. Curr. Microbiol. Appl. Sci.

[11] Nwabor O.F, Dickson I.D, Ajibo Q.C (2015). Epidemiology of *Salmonella* and salmonellosis. Int. Lett. Nat. Sci.

[12] Zhang S, Yin Y, Jones M.B, Zhang Z, Kaiser B.L.D, Dinsmore B.A, Fitzgerald C, Fields P.I, Deng X (2015). *Salmonella* serotype determination utilizing high-throughput genome sequencing data. J. Clin. Microbiol.

[13] Bellhouse D.R (2014). Systematic Sampling Methods. Encyclopedia of Biostatistics.

[14] Soliman E.S, Hassan R.A (2019). Impact of lighting color and duration on productive performance and Newcastle disease vaccination efficiency in broiler chickens. Vet. World.

[15] National Research Council (1994). Nutrient Requirements for Poultry.

[16] Applegate T.J, Angel R (2014). Nutrient requirements of poultry publication:History and need for an update. J. Appl. Poult. Res.

[17] Wei H, Aengwanich W (2012). Biosecurity evaluation of poultry production cluster (PPCs) in Thailand. Int. J. Poult. Sci.

[18] Soliman E.S, Hamad R.T, Ahmed A (2017). Prophylactic and immune-modulatory influences of *Nigella sativa* Linn. in broilers exposed to biological challenge. Vet. World.

[19] Thrusfield M (2005). Veterinary Epidemiology.

[20] Soliman E.S, Hassan R.A (2017). Evaluation of superphosphate and meta-bisulfite efficiency in litter treatment on productive performance and immunity of broilers exposed to ammonia stress. Adv. Anim. Vet. Sci.

[21] Jesudason M.V, Sridharan G, Arulselvan R, Babu P.G, John T.J (1998). Diagnosis of typhoid fever by the detection of anti-LPS and anti-flagellin antibodies by ELISA. Indian J. Med. Res.

[22] Wain J, Hosoglu S (2008). The laboratory diagnosis of enteric fever. J. Infect. Dev. Ctries.

[23] Soliman E.S, Sallam N.H, Abouelhassan E.M (2018). Effectiveness of poultry litter amendments on bacterial survival and *Eimeria* oocyst sporulation. Vet. World.

[24] American Public Health Association, American Water Works Association, Water Environment Federation. (2012) Standard Methods for the Examination of Water and Wastewater.

[25] Kim S.K, Lee J.H (2016). Biofilm modeling systems. Korean J. Microbiol.

[26] Murray P.R, Rosenthal K.S, Pfaller M.A (2015). Medical Microbiology.

[27] Green S.B, Salking N.J (2016). Using SPSS for Windows and Macintosh, Books a la Carte.

[28] Sharma D, Singh N.K, Singh H, Joachim A, Rath S.S, Blake D.P (2018). Discrimination, molecular characterization and phylogenetic comparison of porcine Eimeria spp. in India. Vet. Parasitol.

[29] Chapman H.D, Jeffers T.H (2014). Vaccination of chickens against coccidiosis ameliorates drug resistance in commercial poultry production. Int. J. Parasit.

[30] BAHS (2015). Annual Report, 2014-2015.

[31] Helal A.M, Arafa A, Abdien H.F, Hamed D.M, ElDimerdash M.Z (2017). Avian influenza in liver bird markets in the Suez Canal Region, Egypt. Zagazig Vet. J.

[32] ElMasry I, Elshiekh H, Abdlenabi A, Saad A, Arafa A, Fasina F.O, Lubroth J, Jobre Y.M (2017). Avian influenza H5N1 surveillance and its dynamics in poultry in live bird markets, Egypt. Transbound. Emerg. Dis.

[33] Rothrock M.J, Ingram K.D, Gamble J, Guard J, Cicconi-Hogan K.M, Hinton A, Hiett K.L (2015). The characterization of *Salmonella*
*enetrica* serotypes isolated from the scalder tank water of a commercial poultry processing plant:Recovery of a multidrug-resistant Heidelberg strain. Poult. Sci.

[34] Fasanmi O.G, Odetokun I.A, Balogun F.A, Fasina F.O (2017). Public health concerns of highly pathogenic avian influenza H5N1 endemicity in Africa. Vet. World.

[35] Soliman E.S, Reddy P.G, Sobieh M.A.A, Busby H, Rowe S (2009). Epidemiological surveillance on environmental contaminants in poultry farms. Int. J. Poult. Sci.

[36] Wierup M, Wahlström H, Lahti E, Eriksson H, Jansson D.S, Odelros A, Ernholm L (2017). Occurrence of *Salmonella* spp.:A comparison between indoor and outdoor housing of broilers and laying hens. Acta Vet. Scand.

[37] Ahmed S.T, Bostami A.B.M, Mun H.S, Yang C.J (2017). Efficacy of chlorine dioxide gas in reducing Escherichia coli and Salmonella from broiler house environments. J. Appl. Poult. Res.

[38] Aiyedun J.O, Oludairo O.O, Olorunsola I.D, Daodu O.B, Furo N.A (2018). Effectiveness of biosecurity measures in some selected farms in Kwara State, Nigeria. J. Res. For. Wildl. Environ.

[39] Klosha F, Casteel M, Kump F.W, Klein G (2017). Implementation of a risk-orientated hygiene analysis for the control of Salmonella JAVA in the broiler production. Curr. Microbiol.

[40] Soliman E.S, Sobieh M.A.A, Ahmad Z.H, Hussein M.M, Abdel-Latiff H, Moneim A.A (2009). Seasonal epidemiological surveillance on bacterial and fungal pathogens in broiler farms in Egypt. Int. J. Poult. Sci.

[41] Luyckx K.Y, Van Weyenberg S, Dewulf J, Herman L, Zoons J, Vervaet E, Heyndrickx M, De Reu K (2015). On-farm comparisons of different cleaning protocols in broiler houses. Poult. Sci.

[42] Course C.E (2018). Factors Associated with *Salmonella enterica*
*Escherichia coli* and *Clostridium perfringens* in Broiler Barns during Downtime. Master Thesis.

[43] Soliman E.S, Moawed S.A, Ziaan A.M.G (2016). Assessing cleaning and disinfection regime in a slaughterhouse against carcasses contamination. Adv. Anim. Vet. Sci.

[44] Limbergen T.V, Dewulf J, Klinenberg M, Ducatelle R, Gelaude P, Mendez J, Heinola K, Papasolomontos S, Szeleszczuk P, Maes D (2017). Scoring biosecurity in European conventional broiler production. Poult. Sci.

